# Deep Convolutional Neural Networks for Endotracheal Tube Position and X-ray Image Classification: Challenges and Opportunities

**DOI:** 10.1007/s10278-017-9980-7

**Published:** 2017-06-09

**Authors:** Paras Lakhani

**Affiliations:** Thomas Jefferson University Hospital, Sidney Kimmel Jefferson Medical College, Philadelphia, PA 19107 USA

**Keywords:** Machine learning, Artificial neural networks (ANNs), Radiography, Classification, Artificial intelligence

## Abstract

The goal of this study is to evaluate the efficacy of deep convolutional neural networks (DCNNs) in differentiating subtle, intermediate, and more obvious image differences in radiography. Three different datasets were created, which included presence/absence of the endotracheal (ET) tube (*n* = 300), low/normal position of the ET tube (*n* = 300), and chest/abdominal radiographs (*n* = 120). The datasets were split into training, validation, and test. Both untrained and pre-trained deep neural networks were employed, including AlexNet and GoogLeNet classifiers, using the Caffe framework. Data augmentation was performed for the presence/absence and low/normal ET tube datasets. Receiver operating characteristic (ROC), area under the curves (AUC), and 95% confidence intervals were calculated. Statistical differences of the AUCs were determined using a non-parametric approach. The pre-trained AlexNet and GoogLeNet classifiers had perfect accuracy (AUC 1.00) in differentiating chest vs. abdominal radiographs, using only 45 training cases. For more difficult datasets, including the presence/absence and low/normal position endotracheal tubes, more training cases, pre-trained networks, and data-augmentation approaches were helpful to increase accuracy. The best-performing network for classifying presence vs. absence of an ET tube was still very accurate with an AUC of 0.99. However, for the most difficult dataset, such as low vs. normal position of the endotracheal tube, DCNNs did not perform as well, but achieved a reasonable AUC of 0.81.

## Introduction

One of the goals of a trained radiologist is to accurately describe the presence and position of an endotracheal (ET) tube on chest radiography [1]. There are important consequences of ET tube malposition—a low insertion of the tube into the main stem bronchus can lead to hyperinflation of one lung and pneumothorax, and atelectasis, and hypoxemia of the contralateral non-ventilated lung [2]. Increased mortality and pneumonia have also been reported with low positioning of the tube into the bronchi [3]. As such, there has been interest in using computer-aided detection (CAD) methods to facilitate detection of ET tubes [4–6]. Recent studies using feature extraction and classification with support vector machines have resulted in area under the curves (AUC) of 0.88 and 0.94, respectively, for detection of ET tubes [5, 6].

Given the recent success of deep convolutional neural networks (DCNNs) in regard to image classification [7, 8] on the ImageNet Large Scale Visual Recognition Competition (ILSVRC) [9], there has been interest and some early success in applying them to medical imaging, including cardiomegaly and pleural effusion assessment on chest radiography, lymph node detection on CT, brain segmentation, and assessing diabetic retinopathy [10–14]. Thus, one of the goals of this study is to evaluate its efficacy in assessing the presence and location of ET tubes on chest radiographs.

However, regarding radiography datasets, the number of images needed to train a deep learning algorithm to achieve a reasonable accuracy appropriate for clinical use is still in question. For example, Cho et al. [15] used the GoogLeNet [8] convolutional neural network to classify different axial regions on CT (e.g., head, neck, chest, abdomen, and pelvis), and estimated that about 5000 annotated images per class are required to train a deep neural network to achieve a high (99.5%) target accuracy. However, this number may depend on the difficulty of the training set.

Thus, in this study, we evaluate the efficacy of DCNNs in differentiating subtle, intermediate, and more obvious image differences in radiography. For subtle, we chose ET tube position on single-view chest X-rays—that is, to distinguish between a satisfactory and a low abnormal position. For intermediate, we distinguish between the presence and absence of an ET tube. For more apparent differences, we simply distinguish between chest and abdominal radiographs.

## Methods

This was an IRB-approved, retrospective study using de-identified HIPAA-compliant images obtained from our Picture Archive and Communication System (PACS). Using a search engine that leverages the PACS’s API, multiple sets of images were obtained for this study. This included (a) 300 unique single frontal radiographs that indicated either presence (*n* = 150) or absence of an endotracheal tube (*n* = 150), (b) 300 radiographs indicating a low (*n* = 150) or satisfactory position (*n* = 150) of an endotracheal tube, and (c) 120 radiographs consisting of normal chest (*n* = 60) and normal abdominal radiographs (*n* = 60).

Regarding tube position, a low position was defined as a tube near or below the level of the carina. A satisfactory position was defined as the tube tip 3–7 cm above the carina [1]. High positions of the ET tube were not assessed in this study. All the datasets were verified by two board-certified radiologists, including the author of the radiology report, and by a separate cardiothoracic radiologist (P.L.) after looking at the images independently.

The chest radiographs were saved in Portable Networks Graphics (PNG) format. For classification of chest vs. abdominal radiographs, and presence vs. absence of an endotracheal tube, the entire chest radiograph was used (image size range 2428 × 1810–4238 × 3480 pixels). However, for classification of tube position (low vs. normal), the images were manually cropped to a smaller size that included the supraclavicular region to the mid-heart, and extended bilaterally to the mid-clavicular line. Most of the cropped images ranged from 400 to 600 pixels in either dimension.

The grayscale radiographic images were resized to a 256 × 256 matrix. The images were loaded onto a computer running Ubuntu 14.04 loaded with the Caffe deep learning framework [16], with CUDA 7.5 and cuDNN dependencies for GPU acceleration. The computer contained an Intel i5 3570 k 3.4 gHz processor, 2 TB hard disk space, 32 GB RAM, and an Nvidia GeForce GTX Titan X Maxwell graphics processing unit (Nvidia Corporation, Santa Clara, CA).

The following deep convolutional neural networks were used to classify the images: AlexNet Untrained (AlexNet_U), AlexNet Pre-trained on ImageNet (AlexNet_T), GoogLeNet Untrained (GoogLeNet_U), and GoogLeNet Pre-trained on ImageNet (GoogLeNet_T) [7, 8]. Pre-trained networks were acquired from the Caffe Model Zoo (http://caffe.berkeleyvision.org/model_zoo.html, BVLC, Berkeley, CA). Training was performed for 90 epochs, with a base learning rate of 0.01 for untrained models, and 0.001 for the trained models, using stochastic gradient descent, with binary cross-entropy loss using the softmax function as the last layer. Ninety epochs were determined to be sufficient enough for the training and validation loss to stabilize based on multiple trial runs with half the data for all models. For untrained networks, the initial layer was set to random initialization of weights. For the pre-trained networks, the last fully connected layer was set to random initialization of weights. All the other layers were not frozen, but learned at a slower base learning rate of 0.001 as detailed above.

For the datasets, the images were split into training, validation, and test. The training data were used to teach the artificial neural network, validation data for model selection, and test to assess model accuracy on unforeseen cases. For all of the datasets, 60 cases were held out for test, which was estimated to provide reasonable 95% confidence intervals. For presence vs. absence of the ET tube, 180/300 (60%) images were used for training, 60/300 (20%) for validation, and 60/300 (20%) for test. Similarly, for low vs. normal position of the ET tube, 180/300 (60%) images were used for training, 60/300 (20%) for validation, and 60/300 (20%) for test. For abdominal vs. chest radiography, 45/120 (37.5%) images were used for training, 15/120 (12.5%) for validation, and 60/120 (50%) for test.

### Additional Data Augmentation

All the training datasets underwent random cropping (227 × 227 pixels) and horizontal flipping for data augmentation, which were pre-built options into the Caffe framework. No additional augmentation was performed on the chest vs. abdominal radiography dataset. However, the following additional augmentation techniques were used on the presence/absence and low/normal ET tube training datasets: (a) contrast-adaptive limited histogram equalization (CLAHE); (b) rotations of 90, 180, and 270°; (c) rotations of ± 5°; and (d) affine transformation with rotation of pixels into a different location in both the *x* and *y* directions (“SWIRL”). CLAHE augmentation was performed using ImageJ version 1.50i (National Institutes of Health, USA). Rotated images and affine transformation was performed using XnConvert 1.73 (XnSoft Corp., Reims, France). The additional augmentations for the presence/absence and low/normal ET tube images increased the size of these datasets 12-fold, resulting in 2160 images for training and 720 images for validation. No augmentation was performed on the validation or test datasets.

### Statistical Analysis

All statistical analysis was performed using the pROC package, version 1.7.3, within The R programming language, version 3.3.1 (The R Foundation, Vienna, Austria). Receiver operating characteristic (ROC), area under the curves (AUC), and 95% confidence intervals were determined [17–20]. The DeLong non-parametric method was used to assess for statistical differences among the AUCs. A *P* value of less than 0.05 was considered statistically significant.

## Results

For classification of radiographs (abdominal vs. chest), the AUCs are Alexnet_U (0.996), GoogLeNet_U (0.959), Alexnet_T (1.000), and GoogLeNet_T (1.000) (Table 1). No statistically significant differences were present between these models.

**Table 1:** AUCs of the classifiers for the three different datasets

	Chest/abdomen X-rays	ET tube presence/absence	ET tube low/normal
Untrained AlexNet	0.996 (0.987–1.000)	0.675 (0.537–0.813)	0.741 (0.612–0.870)
Untrained GoogLeNet	0.959 (0.905–1.000)	0.650 (0.505–0.795)	0.748 (0.623–0.873)
Pre-trained AlexNet	1.000 (1.000–1.000)	0.856 (0.758–0.954)	0.791 (0.672–0.910)
Pre-trained GoogLeNet	1.000 (1.000–1.000)	0.989 (0.970–1.000)	0.809 (0.697–0.921)

The numbers in parentheses represent the 95% confidence intervals

For presence vs. absence of the ET tube, the AUCs are Alexnet_U (0.675), GoogLeNet_U (0.650), Alexnet_T (0.856), and GoogLeNet_T (0.989) (Table 1). The pre-trained models for AlexNet and GoogLeNet performed better than the untrained models (*P* = 0.02 and *P* < 0.001, respectively). The best-performing GoogLeNet_T model also performed better than the best-performed AlexNet_T model (*P* < 0.007).

For ET tube position (satisfactory vs. low), the AUCs are Alexnet_U (0.741), GoogLeNet_U (0.748), Alexnet_T (0.791), and GoogLeNet_T (0.809) (Table 1). No statistically significant differences were present between these models.

The best-performing GoogLeNet_T model for presence/absence of ET tube (AUC 0.989) was significantly more accurate than the best-performing GoogLeNet_T model for low/normal ET tube position (AUC 0.809), *P* = 0.002.

**Fig. 1:**
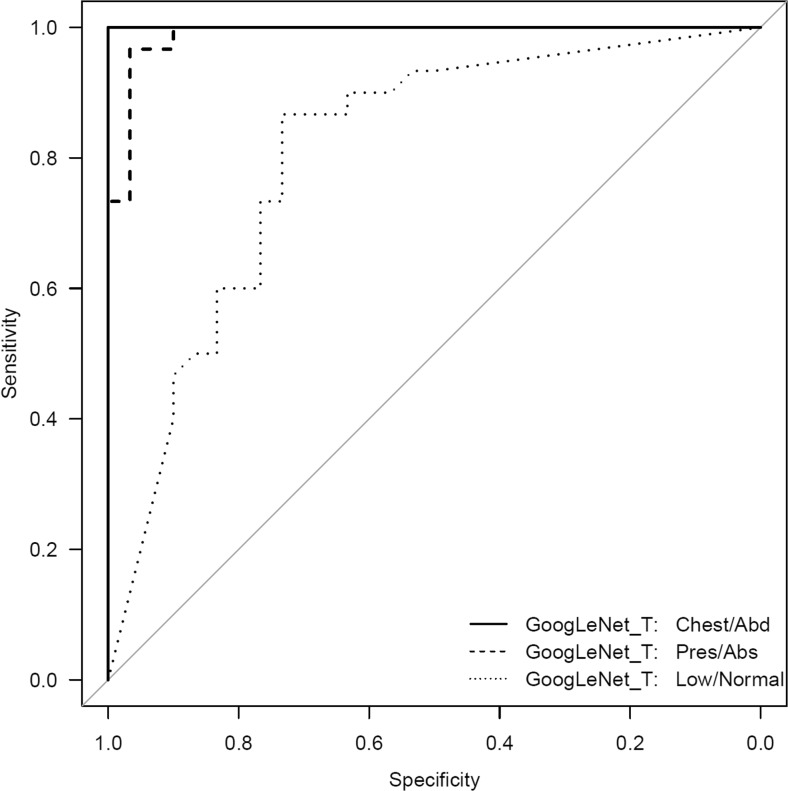
ROC curves of the best-performing classifiers for the three different datasets. The AUC of the chest/abdomen model was the highest at 1.000 (*solid black line*), followed by the presence/absence at 0.989 (*dashed black line*), and low/normal at 0.809 (*dotted black line*)

A comparison of the AUCs of the models without additional augmentation is provided in Table 2. The extra-augmentation resulted in greater AUC values for both AlexNet and GoogLeNet on the ET presence/absence and ET low/normal datasets, but this was only statistically significant for the pre-trained GoogLeNet_T model for ET tube presence/absence dataset (*P* < 0.0001). A comparison of augmentation with shallow and quadrilateral rotations is provided in Table 3. ROC curves for the best-performing models for each of the three datasets are provided in Fig. 1.

**Table 2:** Comparison of models with augmentation and without augmentation for the ET presence/absence and ET low/normal datasets

	ET tube presence/absence	ET tube low/normal
AlexNet_T No Aug	0.771 (0.647–0.895)	0.763 (0.640–0.886)
AlexNet_T Aug	0.856 (0.758–0.954)	0.791 (0.672–0.910)
Significance	*P* = 0.245	*P* = 0.487
GoogLeNet_T No Aug	0.636 (0.494–0.778)	0.713 (0.584–0.843)
GoogLeNet_T Aug	0.989 (0.970–1.000)	0.809 (0.697–0.921)
Significance	*P* < 0.0001	*P* = 0.141
AlexNet_U No Aug	0.669 (0.531–0.807)	0.613 (0.461–0.766)
AlexNet_U with Aug	0.675 (0.537–0.813)	0.741 (0.612–0.870)
Significance	*P* = 0.933	*P* = 0.063
GoogLeNet_U No Aug	0.491 (0.334–0.642)	0.657 (0.513–0.800)
GoogLeNet_U with Aug	0.650 (0.505–0.795)	0.748 (0.623–0.873)
Significance	*P* = 0.216	*P* = 0.328

AUCs and corresponding *P* values are provided

**Table 3:** Augmentation with ±5° and ±90° rotations and corresponding AUC values and *P* values

Model	AUC	Model	AUC
AlexNet_T (±5°)	0.762 (0.635–0.889)	AlexNet_U (±5°)	0.688 (0.551–0.824)
AlexNet_T (±90°)	0.727 (0.596–0.858)	AlexNet_U (±90°)	0.671 (0.534–0.801)
	*P* = 0.615		*P* = 0.787
GoogLeNet_T (±5°)	0.810 (0.699–0.921)	GoogLeNet_U (±5°)	0.642 (0.498–0.785)
GoogLeNet_T (±90°)	0.736 (0.608–0.864)	GoogLeNet_U (±90°)	0.603 (0.455–0.752)
	*P* = 0.163		*P* = 0.668
GoogLeNet_T (±5°) CLAHE & SWIRL	0.834 (0.732–0.935)	AlexNet_T (±5°) CLAHE & SWIRL	0.781 (0.661–0.901)
GoogLeNet_T (±90°) CLAHE & SWIRL	0.989 (0.970–1.000)	AlexNet_T (±90°) CLAHE & SWIRL	0.856 (0.756–0.955)
	*P* = 0.004		*P* = 0.325

The bottom rows also include other augmentation methods, including CLAHE and “swirl” (non-rigid transformation)

**Fig. 2:**
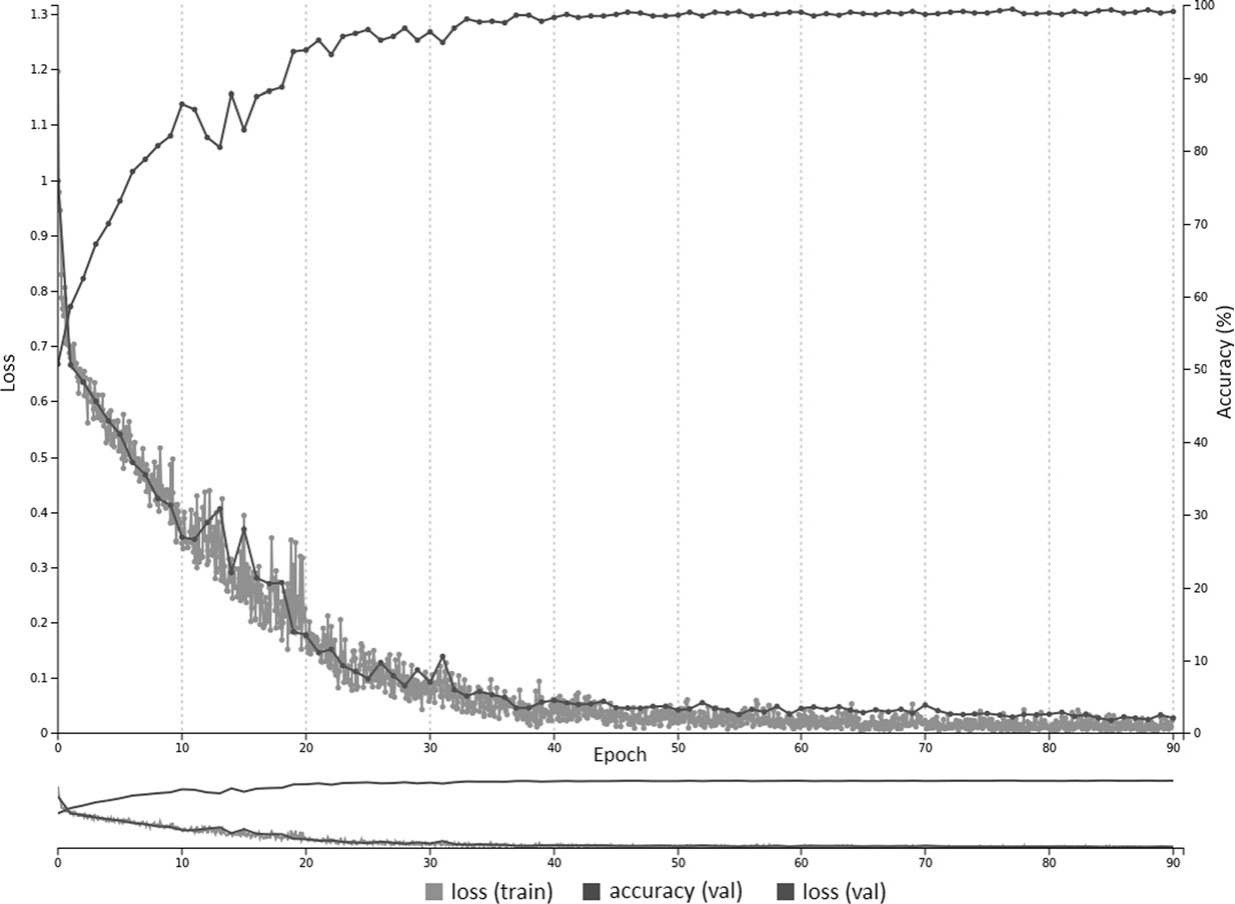
With reduced learning rate of 0.001, there is fast convergence using pre-trained weights on the AlexNet model. Both training and validation loss reduce over the course of training, with accuracy on the validation dataset reaching approximately 99% at 90 epochs

**Fig. 3:**
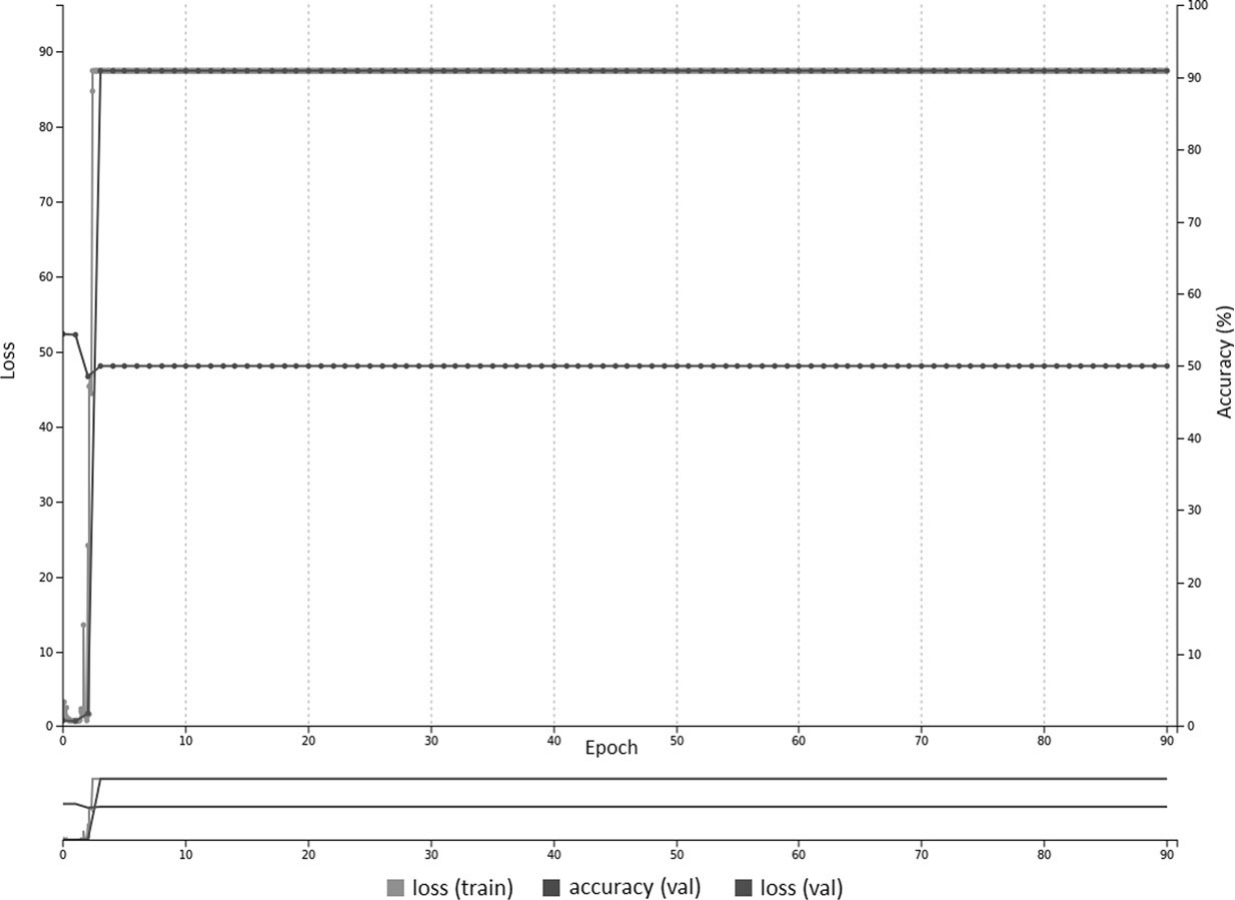
With higher learning rate of 0.01, there is no convergence after 90 epochs using pre-trained weights on the AlexNet model, likely due to large gradients that impact the learning process. The training and validation loss remains high with accuracy constant at 50%

A training curve for the AlexNet_T model is provided in Fig. 2 with a base learning rate of 0.001. Figure 3 is the same pre-trained model but with a higher base learning rate of 0.01, which is the same as untrained networks.

**Fig. 4:**
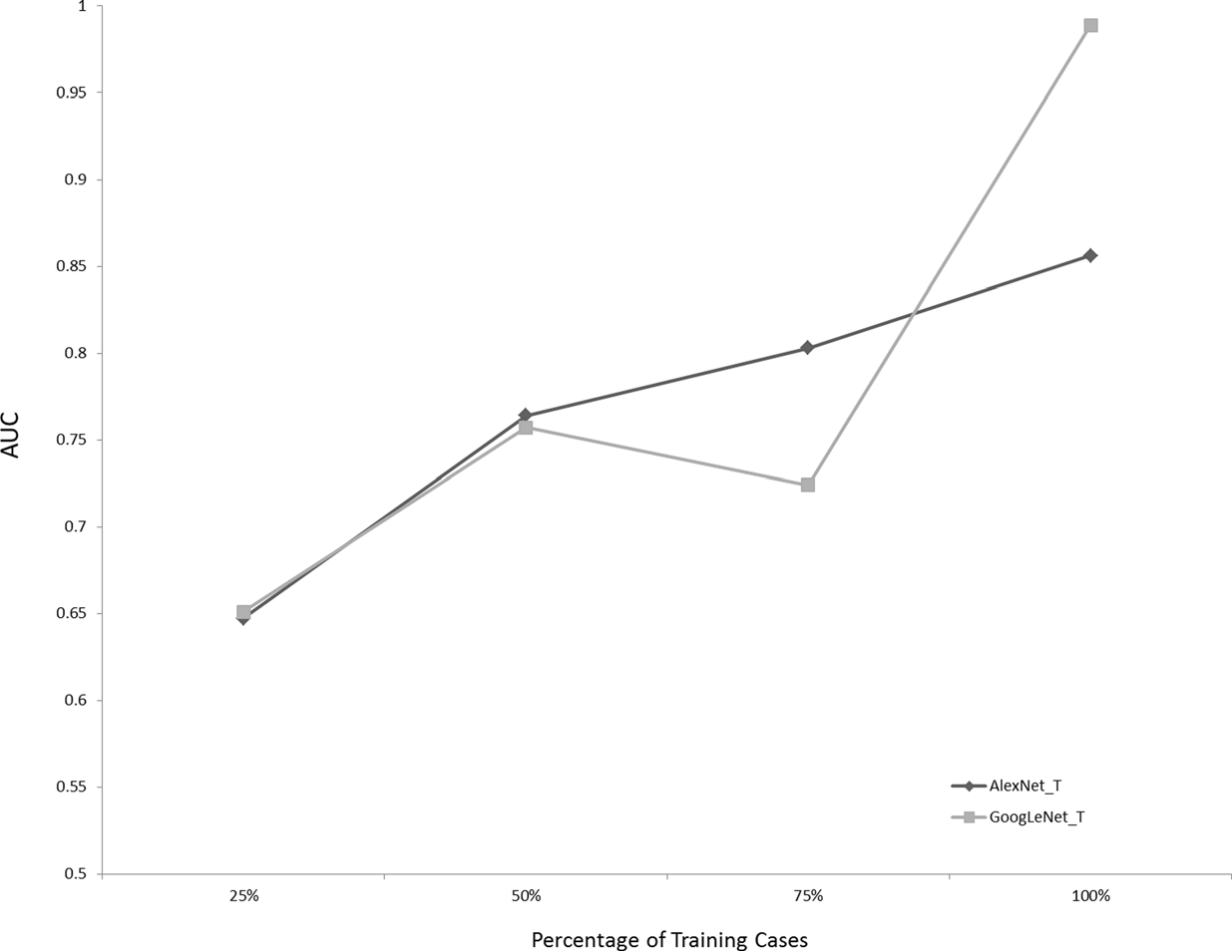
Effect of number of training cases on AUCs for the pre-trained models for the ET presence/absence dataset. Using 100% of training cases resulted in statistically significant higher AUCs than with 25% of the data (*P* = 0.015 and *P* < 0.0001, for AlexNet_T and GoogLeNet_T, respectively)

Figure 4 demonstrates the AUCs of the pre-trained networks for the ET presence/absence dataset, using 25, 50, 75, and 100% of the available training data. Using 100% of the training data resulted in higher AUCs for GoogLeNet_T and AlexNet_T than 25% of the data (*P* = 0.015 and *P* < 0.001, respectively).

## Discussion

Deep convolutional neural networks are a type of artificial neural network that utilizes multiple hidden layers. Convolutional neural networks have been shown to be highly accurate with image classification, which may be due to its ability to represent images at different layers, including edges and blobs at earlier layers, parts of objects at intermediate layers, and whole objects at later layers.

In this study, we explored three datasets with varying levels of difficulty. For example, with the easiest dataset—differentiation of chest vs. abdominal radiographs—both the pre-trained networks AlexNet_T and GoogLeNet_T achieved 100% accuracy, with only 45 training cases and no additional data-augmentation techniques. Moreover, even the untrained networks achieved very high accuracy (Table 1). On the other hand, four times as many training cases and data-augmentation techniques were used for the presence/absence and low/normal ET tube position datasets, resulting in 2160 training images, which is 48 times the number of that used by the chest/abdominal radiography dataset. However, despite this, the best-performing presence/absence and low/normal ET tube models were still not as accurate the chest/abdominal model (Table 1).

This may be explained by the rich set of differences between chest and abdominal radiographs which can be appreciated in almost any aspect of the image. There is also a stark difference in contrast between the lungs, which have a high number of darker pixels, compared to that of the abdomen, which have a larger number of relatively brighter pixels. As such, with datasets containing more ostensible differences, fewer training examples may be needed to develop an accurate model.

**Fig. 5:**
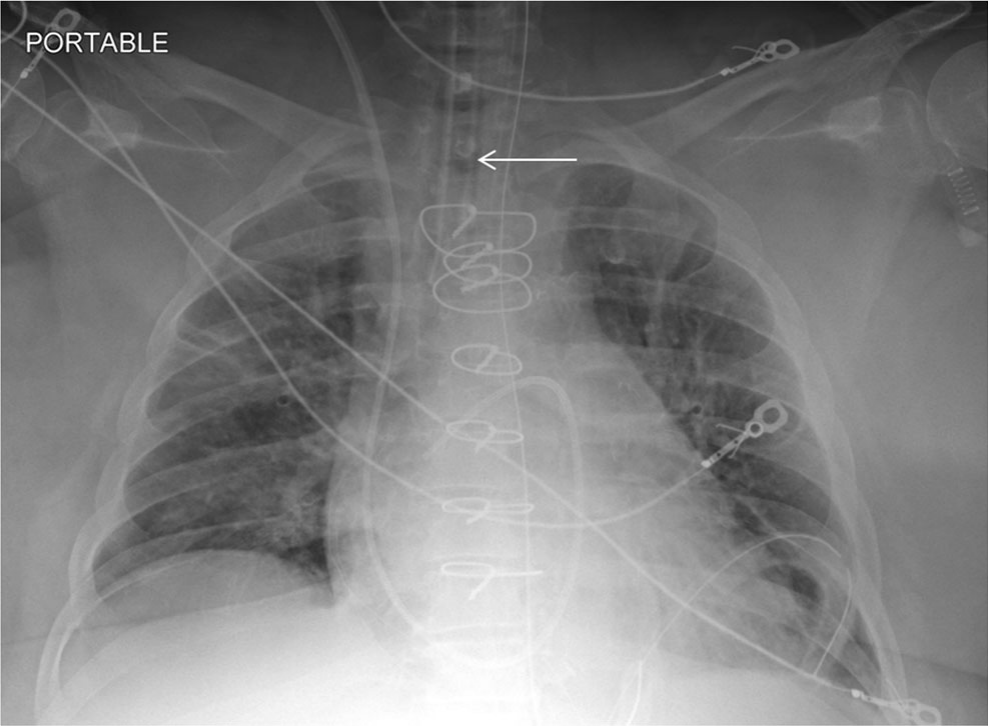
Chest radiograph with ET tube present (*white arrow*)

**Fig. 6:**
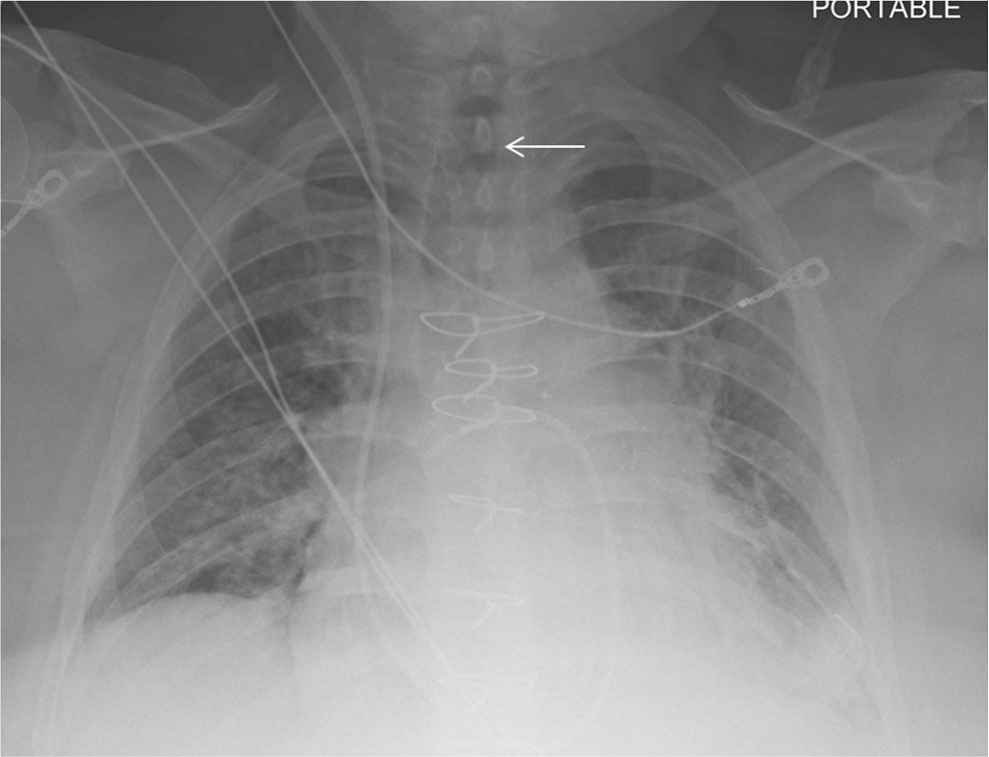
Follow-up chest radiograph on the same patient after ET tube has been removed. The *white arrow* depicts the absence of the ET tube

**Fig. 7:**
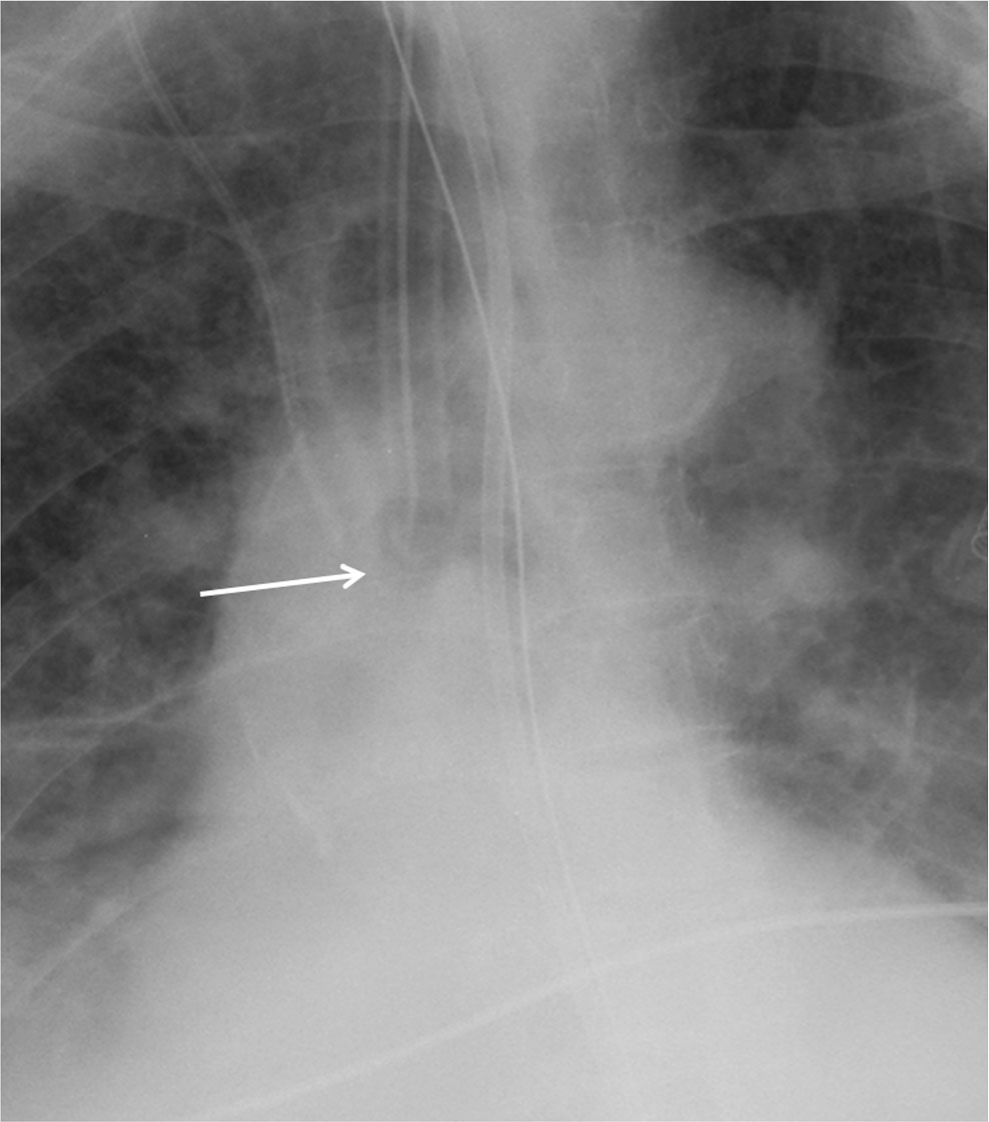
Cropped chest radiograph image centered on the carina. The ET tube tip is low at the orifice of the right main stem bronchus (*white arrow*)

**Fig. 8:**
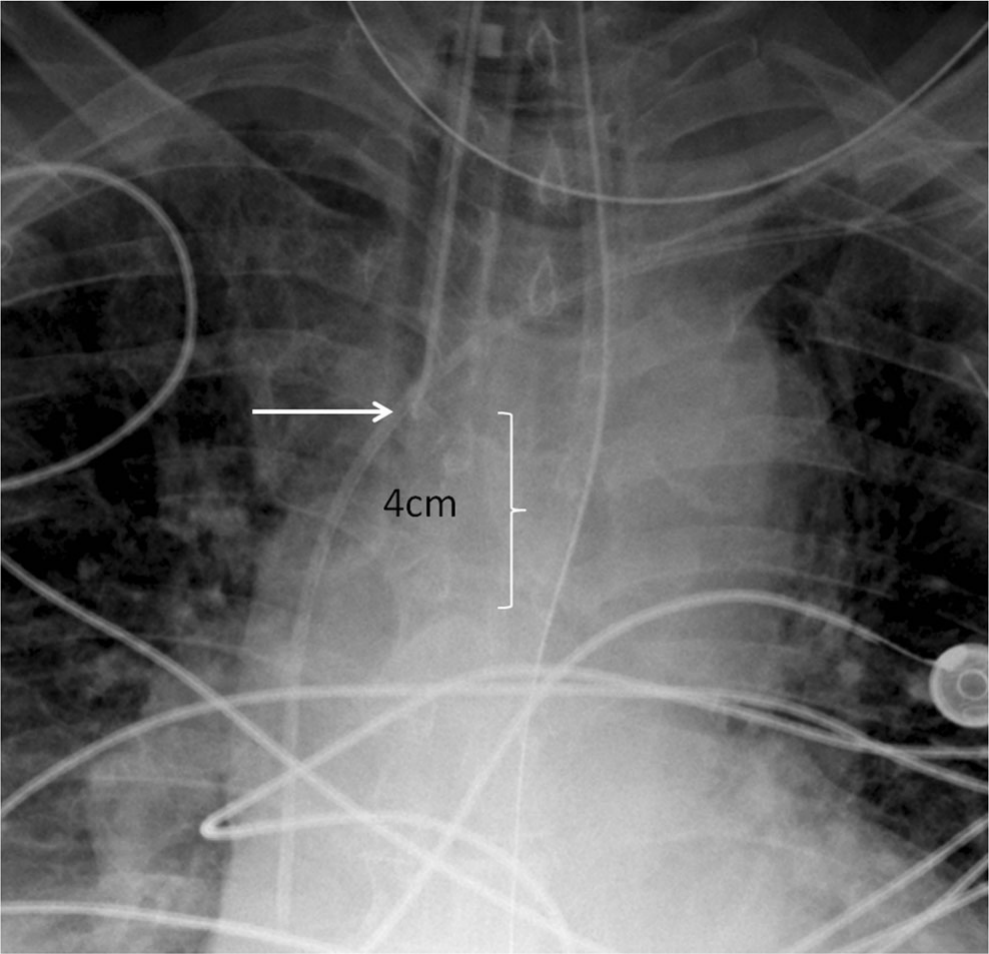
Cropped chest radiograph image shows that the ET tube tip (*white arrow*) is in satisfactory position 4 cm above the carina

On the other hand, differentiating presence and absence of an ET tube can be considered more difficult [21]. This may be because the proportion of the image that changes with the presence or absence of the ET tube is smaller (Figs. 5 and 6), whereas with chest and abdominal radiographs, there are many aspects of the image that are different. However, using pre-trained networks and data augmentation, an AUC of 0.989 was obtained with the best-performing GoogLeNet-T model (Table 1 and Fig. 1). The most challenging dataset was determining low vs. normal position of the ET tube. One explanation is that the carina can sometimes be difficult to identify on portable antero-posterior (AP) chest radiographs, which are often degraded by image noise and have less contrast resolution compared to PA radiographs [21]. This classification task requires assessment of the tube tip as well as the position of the carina. To help the neural network classifiers, these images were manually cropped and centered on the trachea including the bifurcation region (Figs. 7 and 8). One of the limitations is that manually cropping was performed in this study; however, automated cropping centered around the carina would be needed for an automated implementation. Another limitation was that a “high” position of the ET tube was not assessed for this study, which would be worthwhile to consider in future research. The best-performing dataset was a pre-trained GoogLeNet model with an AUC of 0.809 (Table 1 and Fig. 1).

Overall, GoogLeNet performed better than AlexNet for the ET presence/absence and low/normal datasets (Table 1). GoogleNet or Inception V1 is a relatively newer architecture compared to AlexNet, and had a better accuracy on ImageNet (top-5 error rate of 6.7% compared to 15.4% for AlexNet) [7, 8]. This may be explained by the extra depth of GoogleNet, which is 22 layers deep, compared to AlexNet, which is 8 layers deep. The GoogLeNet architecture was able to achieve a deeper architecture and reduce the computational cost of such at the same time by use of the “inception” module. This consisted of smaller 1 × 1 convolutions in parallel to reduce the number of features, before more computationally expensive larger 3 × 3 and 5 × 5 convolutions, sometimes referred to as a “bottleneck” design. As such, GoogLeNet is considered one of the most efficient neural network architectures. This design also provides a mixture of smaller, intermediate, and larger convolutions, which may help integrate information from a wider portion of the image.

It is possible that down-sampling the data to 256 × 256 pixels—which is commonly performed in other deep learning studies [7, 8]—reduced the classifier’s accuracy for both the ET presence and localization tasks. Of the two, the ET tip localization is more dependent on spatial resolution information, and better preservation of resolution may be needed for accurate models. Of course, higher-resolution images require more GPU memory, which may be a limiting factor for some depending on the depth and memory constraints of the neural network. Further research using higher-resolution images would be worthwhile to perform.

Transfer learning using networks pre-trained on non-medical images (ImageNet) performed better than untrained networks (Table 1), in keeping with prior studies [10, 11, 13]. This was only shown to be statistically significant for the presence/absence GoogLeNet_T and AlexNet_T models compared to their untrained counterparts. The first layer of well-trained networks tends to have general features comprised of edges and blobs, as shown with multiple neural network architectures regardless of the type of training data [22]. On the other hand, the last layers are thought to be specific to the training data—in this case, chest radiographs. As such, it makes sense that leveraging pre-trained weights of the initial layers of a well-formed neural network and training mostly on the last layer (set to random initialization of weights) should improve accuracy. In this study, all of the layers were not frozen, but rather set to learn at a slower rate. Rajkomar et al. demonstrated that pre-training with grayscale images from ImageNet resulted in better accuracy than pre-training with color images, when using transfer learning for training with grayscale chest radiographs [11]. However, this case was only true if all the layers were frozen, except for the last layer. Moreover, that study also demonstrated that similar high accuracy can be obtained using models pre-trained on color images if the layers are *not* frozen (fine-tuning of all layers), and using a reduced learning rate as in the case of this study. Figures 2 and 3 show training curves for the pre-trained AlexNet networks for ET presence/absence using base learning rates of 0.001 and 0.01. The curves show that lower base learning rates (e.g., 0.001) are important when utilizing transfer learning, as the pre-trained weights are already well optimized, and higher rates (e.g., 0.01) may result in slower or no convergence.

For the ET presence/absence and low/normal datasets, additional augmentation was done using pre-processed images, which included contrast enhanced with CLAHE, non-rigid deformation, and quadrilateral rotations (90, 180, and 270°). This was to increase the size of the dataset as well as provide more variation in the images to mitigate overfitting. Overall, models with augmentation resulted in higher AUCs compared to those without, although this was only statistically significant for the GoogLeNet_T model (Table 2). Quadrilateral rotations were chosen because occasionally rotated images are accidentally sent by the modality to the reading worklists, and we wanted the DCCNs to handle that potential variation. In addition, it seems more intuitive that shallow rotation angles (e.g., ±5°) would aid training of the network and preempt overfitting, because of relative similarity to original image. However, when combined with other augmentation strategies (CLAHE and non-rigid deformation), and using pre-trained networks, augmentation with quadrilateral rotations had greater accuracy (Table 3), although this was only statistically significant for the GoogLeNet_T model. It is possible that quadrilateral rotations provide a greater difference in the image, potentially reducing overfitting on the test data. However, more research is needed to see if this is true with other datasets.

**Fig. 9:**
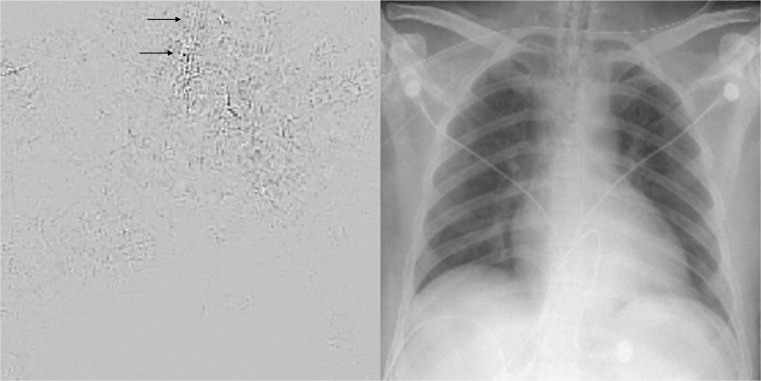
Saliency map on the *left* with corresponding radiograph on the *right*, where an ET tube is present. The saliency map highlights parts of the images that contribute most to the prediction class. The *black arrows* points to the location of the ET tube, which has a visible contribution to the prediction label. However, there are other parts of the image, including edges of ribs and the aortic knob that also contribute to the score for this image. While the network had high accuracy, there is still room for improvement

**Fig. 10:**
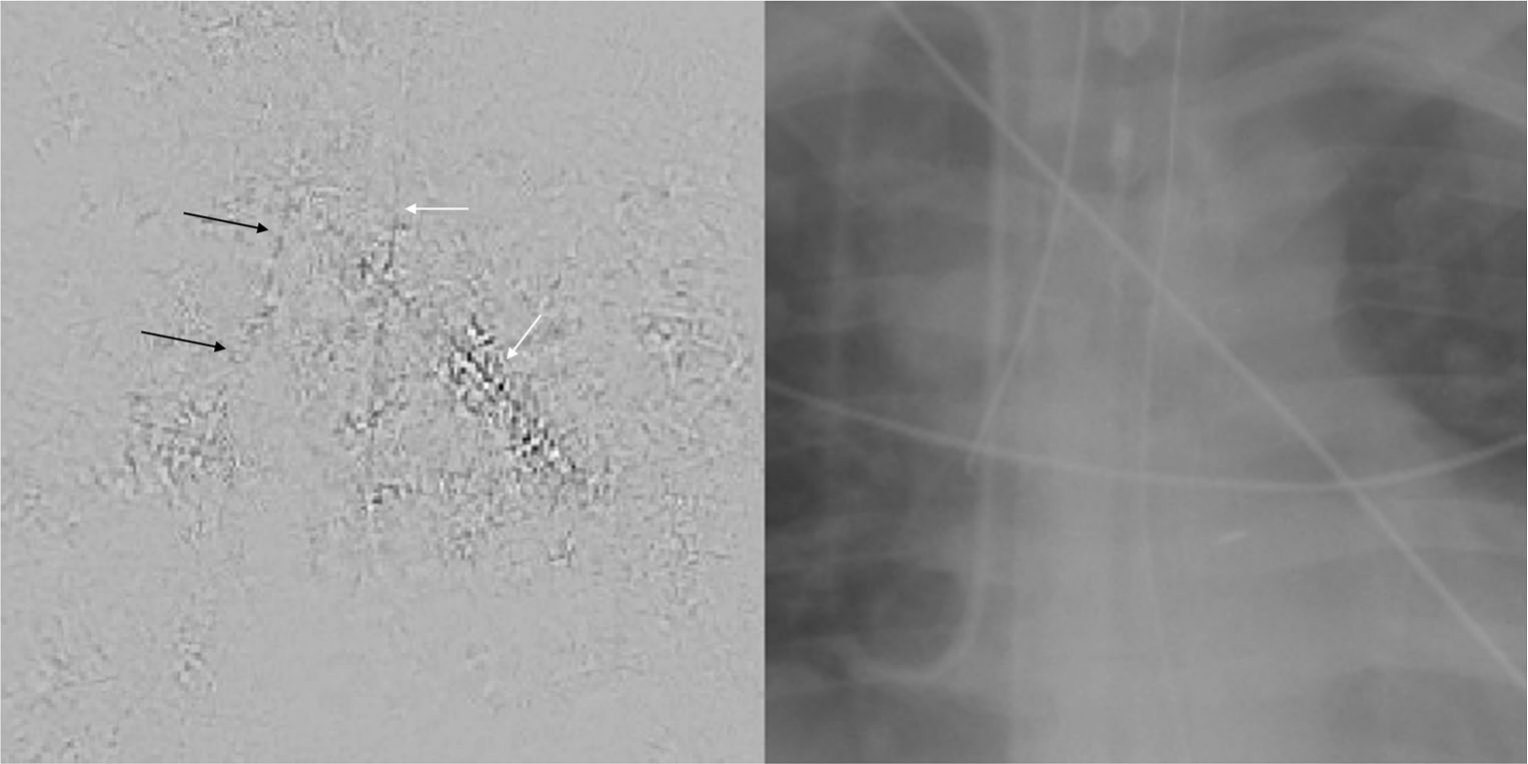
Saliency map on the *left* with corresponding radiograph on the *right* with low position of ET tube in the right main stem bronchus. On the saliency map, the *black arrows* point to the location of the ET tube. The *white arrows* point to an unrelated overlying catheter and an enteric tube. From the saliency map, the ET tube, enteric tube, and overlying catheter all have contributions to the prediction class, indicating that the network is not as well formed and is inferring from parts of the image (enteric tube and overlying catheter) that are not relevant to the prediction label (ET tube is low). This may explain the lower accuracy of this network compared to the others

Deep neural networks can be thought of as functional black boxes, because of the tremendous number of parameters that these networks have. For example, AlexNet has approximately 60 million different parameters and GoogLeNet has 5 million [7, 8]. However, there are strategies to inspect a network and determine the parts of an image that are being activated [23]. One method involves creation of a saliency map, which highlights parts of the network that contribute most to the prediction label, by carrying out an optimization using gradient ascent [23]. Figure 9 is a saliency map for the ET tube presence/absence task, derived from the GoogLeNet_T model. Figure 10 is a saliency map for the ET tube position classification task, also from the GoogLeNet_T model. The maps were created using one back-propagation pass through the DCNN. In these examples, the black parts of the image contribute most to the prediction of the network, as opposed to the light gray background. In Fig. 9, the area of the endotracheal tube at the level of the thoracic inlet has a visible contribution to the prediction class, which lends credence to the model as it is appropriately assessing the correct region. However, there are rib edges and an overlying unrelated catheter that also contribute. Therefore, one could infer that the network has room for improvement. In Fig. 10, the ET tube, enteric tube, and an overlying catheter *all* have contributions to the prediction class, indicating that the network is not as well-formed and is inferring from parts of the image (enteric tube and overlying catheter) that are not relevant to the ground-truth label (ET tube is low). The enteric tube and overlying catheter have similar features with a linear appearance and brighter pixel intensities, which may be confusing the model. It is likely that more training cases could improve this.

One would expect that increasing the number of cases for training should improve accuracy, as deep neural networks have been shown to perform better with larger sample sizes [15]. For example, Fig. 4 shows the AUCs of the pre-trained AlexNet_T and GoogLeNet_T classifiers for the presence/absence data, using 25, 50, 75, and 100% of the total training data. Training with the full dataset resulted in higher AUCs for GoogLeNet_T and AlexNet_T than 25% of the data for example (*P* = 0.015 and *P* < 0.001, respectively; Fig. 4).

To further improve these results, one could consider different types of deep artificial neural networks, pre-processing steps, pre-training on a large sample of radiology images (rather than non-medical images), higher matrix sizes, working with DICOM files directly, or using a combination machine learning techniques.

## Conclusions

Deep convolutional neural networks perform rather well in distinguishing images that have many obvious differences, such as chest vs. abdominal radiographs (AUC = 1.00), and require only a small amount of training data. For more difficult datasets, such as the presence/absence or low/normal position of an endotracheal tube, using pre-trained networks, more training cases and data augmentation can increase accuracy. The best-performing model for classifying presence vs. absence of an ET tube was still very accurate with an AUC of 0.99. However, for the most difficult dataset, such as low vs. normal position of the endotracheal tube, DCNNs did not perform as well, but achieved a reasonable AUC of 0.81.
